# Treatment of Depression With Alcohol and Substance Dependence: A Systematic Review

**DOI:** 10.7759/cureus.11168

**Published:** 2020-10-26

**Authors:** Ahmed M Alsheikh, Maryam O Elemam, Manal El-bahnasawi

**Affiliations:** 1 Medicine, College of Medicine, Almaarefa University, Riyadh, SAU; 2 Pharmacy, Dr. Sulaiman Al-Habib Medical Group, Riyadh, SAU

**Keywords:** depression, alcohol, substance use disorder, dependence, treatment

## Abstract

Although alcohol and/or substance use disorders have been significantly associated with depression, data on the treatment outcomes of depression in this patient population are still scarce, especially among the higher risk of resistance to treatment. This study examines the management outcomes of depression in patients with alcohol and substance dependence during the last decade by searching the medical literature. The literature was searched through Medline, PsycInfo, Embase, and Ovid database from 2010 to 2020. Searching terms included were a combination of ‘’treatment’’ AND ‘’Depression’’ AND ‘’alcohol’’ OR “substance abuse". A total of 617 articles were retrieved. After this, original articles investigating depression treatment outcomes in patients with alcohol or substance use disorders or both were selected. Following the exclusion of review studies and including only original research studies, 23 articles appeared. We selected eight articles as eligible, covering a total of 132,373 patients with depression and either alcohol dependence or substance use disorder. Anti-depressants (mainly selective serotonin reuptake inhibitors) combined with psychotherapy and alcohol or substance abuse treatment represent the best treatment modality for depression in this clinical setting. In conclusion, patients with alcohol or substance dependence usually suffer from treatment-resistant depression. However, the treatment of depressive symptoms would help in substance or alcohol abstinence and reduce recurrent substance abuse.

## Introduction and background

Substance use disorder (SUD) is a condition that is prevalent in all age groups at all socio-economic levels [1]. It has been defined as using a psychoactive agent, which results in high levels of stress and functional disabilities [2]. This disorder has been reported as a primary factor for disability globally. It is also a contributor to a wide range of psychological and physical disorders, most commonly severe depression [3]. It has been estimated that up to 40% of patients with depression have a history of SUD or alcohol dependence during their lifetime [4]. However, only 19% of these patients seek medical help for themselves, where most of them were alcohol dependence [4]. Despite the availability of information on the correlation between depression and SUD and/or alcohol dependence, their causality is still controversial [5]. The association between substance use and depression has been specified more significantly for some substances, including alcohol, cannabis, and opioids. Some data are also available on stimulant agents and benzodiazepines [6].

Generally, depression is a significant contributor to disability all over the world. It can lead to life-threatening complications either psychologically or physically, most remarkably suicide [7,8]. Despite the advances in the development of anti-depressants' drugs, the treatment success rate is usually low, not crossing 50% with combined anti-depressants. This percentage is even lower with alcohol and substance abuse [9]. The treatment of depression associated with alcohol or substance abuse has shown high resistance and therapy failure [10]. Moreover, a subset of patients may even suffer from worsened depression symptoms. Accordingly, some studies evaluated the combination of pharmacological treatment with psychotherapy in this patient population [11]. Also, other studies have evaluated the correlation between abusing particular substances and the outcomes of depression treatment. However, the available data are debatable [12]. This systematic review aimed to examine the literature for depression treatment outcomes in patients with alcohol and/or SUDs.

## Review

Methodology

This systematic review was conducted based on the PRISMA (Preferred Reporting Items for Systematic Reviews and Meta-Analyses) checklist recommendations for systematic review and meta-analysis [13]. This systematic review was performed by searching electronic databases to include eligible trials from 2010 till September 2020 in four databases, including Medline, PsycInfo, Embase, and Ovid.

Search strategy

Searching terms included ‘’treatment’’ AND ‘’Depression’’ AND ‘’alcohol’’ OR “substance abuse”. All the titles and abstracts that appeared from this search were reviewed thoroughly to prevent missing any eligible articles. We included the results of only original research articles investigating depression treatment outcomes in patients with alcohol use disorder or SUD or both. Selected trials mentioned the condition under investigation, whether it is depression with abused substance or alcohol dependence. Additionally, all studies from different countries were eligible. Only studies published in English were classified as related articles, which can be further evaluated in the second step.

Eligibility criteria

**Figure 1:**
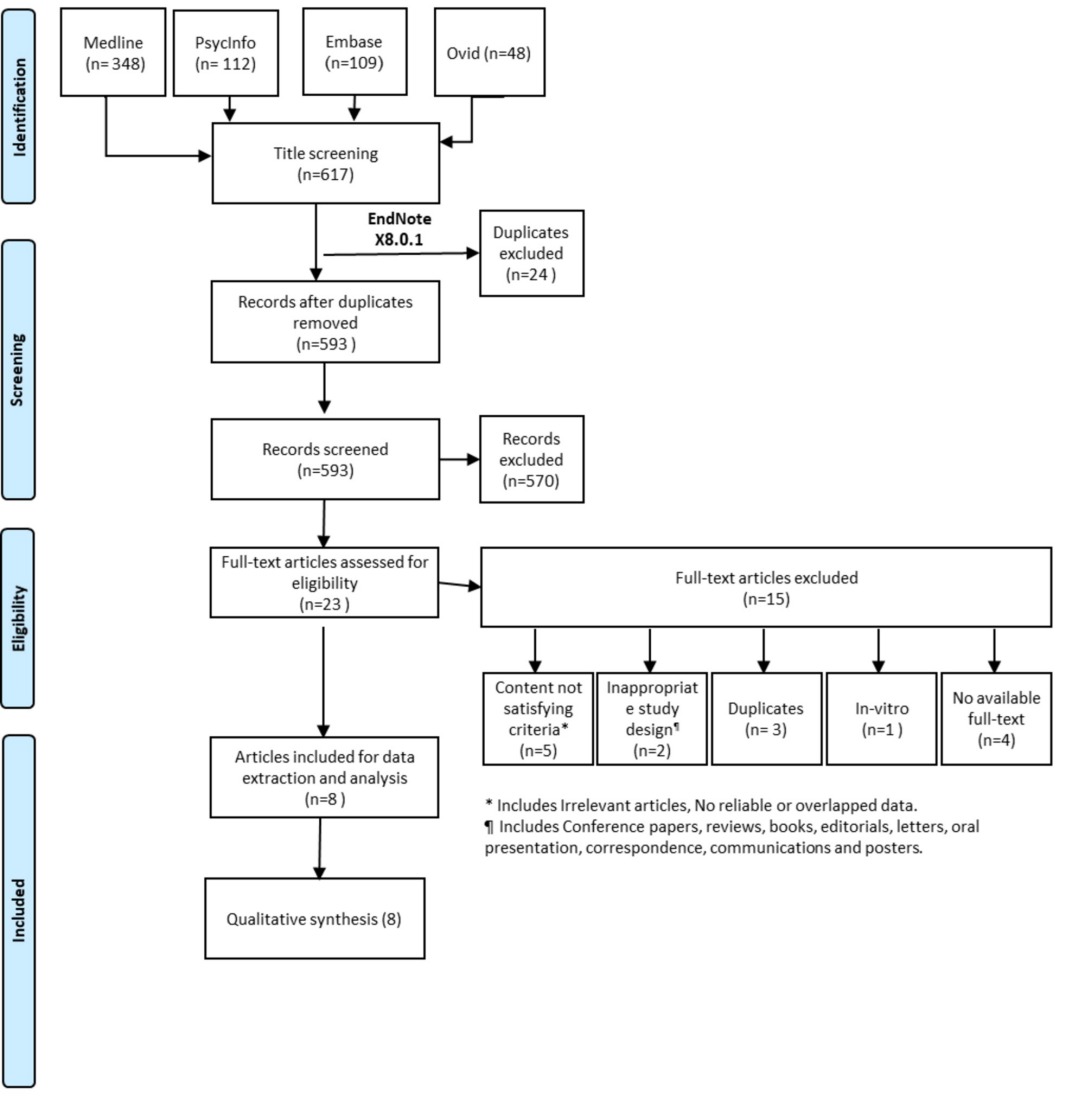
PRISMA flowchart of the search and screening process PRISMA, Preferred Reporting Items for Systematic Reviews and Meta-Analyses

Abstracts were examined manually to choose sufficient, clear, and adequate abstracts. The inclusion criteria were mentioning data on the outcomes of depression treatment in either SUD of different agents or alcohol dependence or both. Afterward, we evaluated references of the selected trials to identify any related articles. Finally, we gathered the required data sets from the final record of eligible articles and summarized. We excluded articles that were review studies, those with overlapped or incomplete data, in vitro studies, and unavailability of full-text articles or inappropriate study design (Figure 1).

Data review and analysis

We included a preliminary review and used a specially designed excel sheet for data extraction. Next, we selected data from eligible studies and then revised them through the Excel sheet. We reviewed any articles published by one research group investigating similar variables for any possible duplication. Cochrane, a quality assessment tool, was also used to evaluate the included studies [14].

Results

After searching the abstracts and checking for the eligibility criteria in identified potential abstracts, eight articles were regarded as a potential for inclusion in the present systematic review published between January 2010 and September 2020, covering 132,373 patients with depression and either alcohol dependence or SUD.

Out of the eight included studies [15-22], only one study had a retrospective design [16], whereas the remaining seven studies were prospective, where one study was a case-control study [15], five studies were randomized controlled studies [17,19-22], and one study was a prospective cohort study [18].

Regarding the type of abuse, alcohol dependence with depression was evaluated in six studies [16-18,20-22] either alone or with other abused substances. Opioids were also examined in two studies [15,18], and cannabis was examined in two studies [18,20] (Table 1).

**Table 1:** Table of included studies AUD, alcohol use disorder; CBT-D, cognitive-behavioral treatment for depression; OST, opioid substitution therapy; RR, residential rehabilitation; RTC, relaxation training control; SUD, substance use disorder

Author(s)	Year	Study design	Sample size	Type of abuse	Objective	Result
Brenner et al. [15]	2020	Case-control study	121,669 (national registry)	Opioids and sedatives	To investigate the association between SUD and treatment-resistant depression	SUD during or ≤ 180 days before starting anti-depressant treatment was associated with almost double the risk for treatment-resistant depression. Increased treatment-resistant depression risks were found ≤ 180 days before starting treatment, especially opioids.
Albrecht et al. [16]	2020	Retrospective cohort	9,581	Alcohol dependence disorder	To evaluate the risk of traumatic brain injury in alcohol-dependent patients treated for depression	Female sex, diagnosis of Alzheimer's disease, and anxiety were associated with a higher risk of traumatic brain injury in alcoholic patients. Longer anti-depressants' duration was correlated with decreased risk of newly diagnosed anxiety, insomnia, and substance dependence disorder.
Jordans et al. [17]	2019	Randomized controlled study	162	Alcohol dependence disorder	To evaluate the outcomes of psychological treatments for depression and AUD delivered by community-based counselors	Adding a psychological treatment delivered by community-based counselors for alcohol-dependent patients treated from depression increases treatment success for depression compared with conventional services provided by primary health workers after one year of follow-up.
Anand et al. [18]	2019	Prospective cohort	263	Cocaine, opiates, cannabis, alcohol, and hallucinogens	To evaluate the effects of substance use on the treatment of depressive symptoms	Depressive symptoms were significantly correlated to the frequency of substance use and the type of the substance. Patients with opioid abuse showed more treatment-resistant depressive symptoms compared to cannabis (p < 0.01).
Ross et al. [19]	2016	Randomized controlled trial	200	Comorbid SUD	To determine the efficacy of a modified version of psychological treatment to reduce depression symptoms and substance dependence in RR and OST	The association between depression and substance dependence has been well documented, yet practical and effective treatments are scarce. Psychological treatment, along with anti-depressants, can improve depression symptoms in patients with SUD.
Kay-Lambkin et al. [20]	2012	Randomized controlled trial	163	Alcohol and cannabis	To evaluate clinician-assisted computerized psychological treatment for depression and alcohol/other drug use comorbidity in rural and urban communities	Patients were significantly older and attended significantly more treatment sessions. Computerized treatment was associated with significantly greater alcohol use reductions relative to face-to-face cognitive behavioral therapy/motivational interviewing and supportive counseling, showing high benefit.
Brown et al. [21]	2011	Randomized controlled trial	165	Alcohol dependence	To evaluate the outcomes of CBT-D compared with an RTC in alcohol-dependent patients with significant depressive symptoms	There were significant improvements in depression symptoms and alcohol use over time for all participants. CBT-D showed significantly reduced depressive symptoms, as measured by the Beck Depression Inventory, after six weeks of follow-up. However, there were non-significant differences at any other follow-up.
Pettinati et al. [22]	2010	Randomized controlled trial	170	Alcohol dependence	To evaluate the efficacy of com­bining approved medications for depres­sion (sertraline) and alcohol dependence (naltrexone) in treating patients with both disorders	Sertraline combined with naltrexone produced a higher alcohol abstinence rate (53.7%) and demonstrated a longer delay before relapse to heavy drinking than the naltrexone-sertraline placebo with less tendency to develop depression.

Discussion

Depression is a common psychiatric disorder that can occur at different age groups [6]. Although there are multiple medications used for managing depression, treatment success is usually low, especially in patients with alcohol dependence or SUDs [9]. Hence, some studies have investigated different treatment modalities and examined depression treatment outcomes in these patients. The present review investigated the outcomes of depression treatment in patients with alcohol dependence or substance use. The present review demonstrated that pharmacological treatment alone might not lead to sufficient outcomes of depression treatment in patients with alcohol dependence or SUDs.

Furthermore, these patients usually suffer from treatment-resistant depression. Accordingly, this usually requires a combination therapy and most properly combining pharmacological therapy with psychotherapy. Cognitive-behavioral therapy for depression (CBT-D) has also shown promising outcomes. Additionally, combined anti-depressants (mainly selective serotonin reuptake inhibitors [SSRI]) combined with alcohol dependence medication (naltrexone) can improve treatment outcomes.

Both alcohol dependence and SUD were examined in this review. As for depression co-existing with alcohol dependence, Jordans et al. [17] evaluated the use of psychotherapy combined with anti-depressants for patients with depression and alcohol dependence. They showed that psychotherapy addition would improve treatment success rates, especially when delivered by community-based counselors, after one year of follow-up.

Similarly, Kay-Lambkin et al. showed that psychotherapy could significantly reduce alcohol use in patients with depression and alcohol dependence. Additionally, they reported that patients with higher treatment success rates were significantly older and attended more sessions. It is worth mentioning that the sessions examined by Kay-Lambkin et al. were computerized and had a reasonable acceptance rate [20]. From the included study, 4% preferred computer-delivered treatment, which was associated with significantly greater alcohol use reductions relative to face-to-face cognitive behavioral therapy/motivational interviewing and supportive counseling.

Furthermore, in a large study by Albrecht et al., the risk of traumatic brain injury was evaluated in patients with alcohol dependence and depression. The study showed that females, patients with anxiety, or those with Alzheimer’s disease were at a higher risk of traumatic brain injury. However, use of anti-depressants significantly reduced the incidence of anxiety, insomnia, and substance abuse in these patients [16].

Also, the role of psychotherapy in these patients was examined by Brown et al., who evaluated the use of CBT-D in patients with severe depression co-existing with alcohol dependence. It showed that CBT-D had shown significant improvement in depressive symptoms at the beginning of treatment; however, the improvement was non-significantly different after following up [21].

On the other hand, Pettinati et al. illustrated that a combination of sertraline, an SSRI, with naltrexone as a treatment for alcohol dependence would result in higher alcohol discontinuation rates and a significant reduction in depression symptoms, with acceptable incidence of side effects [22]. As for depression co-existing with SUD, opioids were the most commonly studied agents. Brenner et al. showed that substance abuse could lead to a doubled risk of treatment-resistant depression than non-abusers. Furthermore, treatment resistance was more significant in opioids abusers [15].

These findings were also confirmed by Anand et al., who also examined cocaine, cannabis, alcohol, and hallucinogenic agents. Additionally, their study demonstrated a significant correlation between the severity of depression symptoms and the frequency of administration of the abused substance and its type [18].

However, the included studies had some limitations. Some of those studies did not identify the substance included in their studies, making the outcomes to all types of abused substances unsupportive in that area. Additionally, only SSRI was examined in combination with medications for alcohol dependence. Consequently, this drives the requirement for future studies that examine other anti-depressants with a different mechanism of action in this clinical setting.

## Conclusions

Patients with alcohol dependence or SUD usually suffer from treatment-resistant depression, particularly patients abusing opioids. Combined psychotherapy with anti-depressants and dependence medications can result in best patient outcomes, where SSRI use was commonly studied. Interestingly, telecommunication and computer-based sessions had a higher effect than face-to-face sessions. As a result, such methods should be utilized further with future programs along with the combined therapy approach. Future studies are needed to assess the role of other anti-depressants combined with psychotherapy for patients with alcohol dependence and SUDs as well as study it within the computerized setting.
